# Secreted heat shock protein 90 promotes prostate cancer stem cell heterogeneity

**DOI:** 10.18632/oncotarget.14252

**Published:** 2016-12-27

**Authors:** Krystal D. Nolan, Jasmine Kaur, Jennifer S. Isaacs

**Affiliations:** ^1^ Department of Cell and Molecular Pharmacology, Medical University of South Carolina, Hollings Cancer Center, Charleston, SC, USA

**Keywords:** Hsp90, cancer stem cells, prostaspheres, epithelial to mesenchymal transition (EMT)

## Abstract

Heat-shock protein 90 (Hsp90), a highly conserved molecular chaperone, is frequently upregulated in tumors, and remains an attractive anti-cancer target. Hsp90 is also found extracellularly, particularly in tumor models. Although extracellular Hsp90 (eHsp90) action is not well defined, eHsp90 targeting attenuates tumor invasion and metastasis, supporting its unique role in tumor progression. We herein investigated the potential role of eHsp90 as a modulator of cancer stem-like cells (CSCs) in prostate cancer (PCa). We report a novel function for eHsp90 as a facilitator of PCa stemness, determined by its ability to upregulate stem-like markers, promote self-renewal, and enhance prostasphere growth. Moreover, eHsp90 increased the side population typically correlated with the drug-resistant phenotype. Intriguingly, tumor cells with elevated surface eHsp90 exhibited a marked increase in stem-like markers coincident with increased expression of the epithelial to mesenchymal (EMT) effector Snail, indicating that surface eHsp90 may enrich for a unique CSC population. Our analysis of distinct effectors modulating the eHsp90-dependent CSC phenotyperevealed that eHsp90 is a likely facilitator of stem cell heterogeneity. Taken together, our findings provide unique functional insights into eHsp90 as a modulator of PCa plasticity, and provide a framework towards understanding its role as a driver of tumor progression.

## INTRODUCTION

Metastasis is the major cause of cancer associated mortality. A prevailing view is that this dissemination and ensuing proliferation of cancer cells at distal sites is executed by a small population of cancer stem-like cells (CSCs) that harbor regenerative potential and tumour initiating properties. Although tumour initiating or CSCs were first identified in leukemia more than two decades ago [1], subsequent studies have recognized the presence and importance of these cells in a variety of tumor settings, including breast and prostate cancers [2, 3]. Numerous reports suggest that specific CSC-associated gene targets correlate with the self-renewal and metastatic potential of cancer cells [4–6]. Within the prostate, the existence of epithelial stem cells was first illustrated by Isaacs et al., wherein they established the ability of stem-like cells within the rat prostate to regenerate after castration-induced atrophy [7]. Furthermore, this regenerative population is enriched for stem-like surface markers [8], supporting the premise that, within a cancer context, distinct cell populations endowed with stem-like properties are essential for tumor regrowth.

The epithelial to mesenchymal transition (EMT) developmental pathway has been causally linked with increased tumor invasion, metastasis and therapeutic resistance [9–12]. EMT activation also notoriously increases cellular plasticity [13, 14], along with cell populations endowed with stem-like properties [15], a feature that promotes tumor chemoresistance and contributes to treatment failure [10, 13, 16, 17]]. Within the context of prostate cancer (PCa), EMT activation has been linked with the development of cell populations with stem cell signatures [18]. This has particular significance for PCa treatment, given that cancer lethality is primarily due to tumor recurrence following development of castrate resistant prostate cancer (CRPC) [19]. Of further relevance, EMT activation has been observed in the prostate following androgen-deprivation therapy [20], and PCa tumor cells with diminished expression of Androgen Receptor (AR) have been characterized as harboring both mesenchymal and cancer stem cell (CSC) properties, including tumor initiation [21, 22].

Heat-shock protein 90 (Hsp90), a highly conserved molecular chaperone, is frequently upregulated in tumors [23], and has been an attractive molecular target for pharmacological intervention [24]. Despite this clinical interest, a potential role for Hsp90 as a regulator of CSCs has not been well studied. A handful of reports implicate Hsp90 in maintaining the cancer stem phenotype [25–27], which may be due in part to its physical interaction with a cohort of CSC facilitators [28–30]. However, in non-malignant conditions, Hsp90 inhibition has been shown to protect the stem cell niche [31], indicating the possibility of a context dependent role for the chaperone.

In addition to its intracellular localization, Hsp90 has also been reported in the extracellular space, a trend frequently observed in tumor models. This extracellular Hsp90 (eHsp90) may be found either in a secreted form or on the cell surface [32, 33]. Although the functions of eHsp90 are not well defined, its targeting across multiple tumor models via use of impermeant blocking antibodies or nonpermeable small molecules attenuates tumor cell motility and invasion *in vitro* [34, 35], and blocks invasion and metastasis *in vivo* [36–39], as reviewed [33], supporting a unique role for eHsp90 in tumor progression. We have reported that eHsp90 enhances cellular motility, invasion, and tumorigenicity in prostate cancer models, which may be due to the ability of eHsp90 to initiate EMT events [40, 41]. Given the link between EMT and stemness, and the ability of eHsp90 to modulate EMT events and tumor aggressiveness, we investigated the possibility that eHsp90 may influence CSCs within PCa.

We herein report a novel function for eHsp90 as a facilitator of cancer stemness, a premise confirmed by utilization of several well-established assays designed to assess cancer stem-like properties. We demonstrate the ability of eHsp90 to upregulate a cohort of stem-associated markers. We additionally demonstrate that eHsp90 promotes self-renewal, relevant for tissue regeneration, and prostasphere growth, indicative of the anchorage-independent growth associated with metastatic propensity [42]. Of additional clinical relevance, eHsp90 increased the side population that is typically correlated with a chemoresistant phenotype [43]. Intriguingly, tumor cells with elevated surface eHsp90 exhibited a marked increase in stem-like markers coincident with expression of the EMT effector Snail, indicating that surface eHsp90 may enrich for a unique CSC population. Finally, our collective analysis of putative effectors modulating the eHsp90-dependent CSC phenotype supports the notion that eHsp90 is a facilitator of stem cell heterogeneity. Taken together, our findings highlight a paradigm whereby eHsp90 orchestrates molecular and functional events to promote PCa plasticity and tumor progression.

## RESULTS

### Hsp90 secretion promotes self renewal and expression of stem-like gene targets

**Figure 1:**
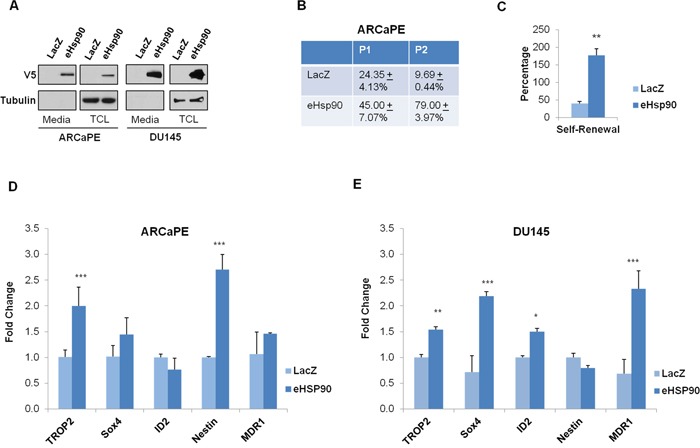
Hsp90 secretion promotes self-renewal and expression of stem-like gene targets **A**. ARCaPE and DU145 prostate cancer cells were stably transduced with either a control (LacZ) plasmid or an expression construct directing the extracellular secretion of Hsp90 (eHsp90). Protein from either total cell lystates (TCL) or conditioned media was evaluated for V5-tagged eHsp90 expression. **B**. Percentage of spheres formed by ARCaPE-LacZ and ARCaPE-eHsp90 as defined by the total number of spheres generated divided by the number of initial wells seeded with single cells from passages 1 and 2 (P1 and P2) in 96 well ultra-low attachment plates. Following 10-12 days, productive self-renewal was assessed by observation of a minimum of 5 cells per well. **C**. Graphical representation of the self-renewal potential of ARCaPE, defined by the percentage of P2 spheres divided by the percentage of P1 spheres. **D, E**. Total RNA was isolated from ARCaPE (D) or DU145 (E) stably transduced with either the LacZ control plasmid or the eHsp90 expression plasmid, and expression of the indicated stem-like targets was assessed by qPCR. All statistics were performed using the Student's t-test. * = p<0.05, ** p<0.01.

We have previously reported a model for directed secretion of Hsp90, whereby Hsp90 alpha is fused to a secretion peptide that facilitates its extracellular localization [40]. We demonstrated that enforced Hsp90 secretion was sufficient to induce EMT events in minimally tumorigenic ARCaPE PCa cells [40]. In this study, we sought to evaluate the effects of eHsp90 in an expanded prostate cancer cell cohort. DU145 is an aggressive androgen independent prostate cancer cell line derived from metastatic tissue [44]. We had previously shown that targeting eHsp90 with the small molecule inhibitor non-permeable geldanamycin (NPGA) attenuated mesenchymal features in DU145 [45]. In this study, we evaluated the molecular and functional effects of enhanced eHsp90 via stable transduction with a lentiviral construct encoding a secreted version of V5-tagged Hsp90. As shown (Figure 1A), the exogenous V5-tagged Hsp90 protein is detected in both lysate and conditioned media fractions derived from transduced ARCaPE and DU145, while it is absent in the corresponding matched LacZ controls. This result confirms that Hsp90 is being secreted in these cell types, therefore validating the utility of these cell models.

Given our prior work indicating that eHsp90 may modulate EMT events [40], and the well-known link between EMT plasticity and stem-like features [9, 14, 16], we explored the possibility that eHsp90 may affect stem-like features in prostate cancer models. A number of functional and molecular assays have been used to identify distinct stem-like populations in cancer cells. We first assessed whether eHsp90 may promote self renewal, a property of cancer cells to proliferate and repopulate a tumor, indicative of the general ability of a cancer cell to proliferate and repopulate a tumor. Following an initial round of growth under suspension conditions (P0), single cells were seeded into low-attachment plates (P1 generation). As shown (Figure 1B), ARCaPE-eHsp90 cells exhibited nearly a two-fold increase in sphere-forming capacity relative to the matched ARCaPE-LacZ control cells when progressing from P0 to P1 passages. Moreover, the sphere forming ability was increased by almost 8-fold when cells from the P2 generation were tested in this assay. This marked increase is in part due to the reduced spheroid efficiency of control (ARCaPE-LacZ) cells (from P1 to P2), and to the enhanced spheroid efficiency of ARCaPE-eHsp90 during similar passaging conditions. Moreover, eHsp90 increased self-renewal over 3-fold (Figure 1C), defined as the capacity to form spheres at P2 generation relative to the P1 stage. The growth of DU145 was minimal under similar conditions and therefore, it was not possible to achieve consistent results with this model.

We next evaluated whether Hsp90 secretion influenced the expression of stem-like gene targets in the ARCaPE and DU145 isogenic models. As shown (Figures 1D, 1E), both Trop2 and Nestin were significantly increased in ARCaPE-eHsp90 relative to LacZ control cells, while Trop2, ID2, and MDR1 (multidrug resistance 1) were increased in DU145-eHsp90 relative to its LacZ control. Although MDR1 is not a specific stem cell marker, this gene, and its family members, are frequently detected in stem cell populations in a number of cancer models [46] including prostate [47]. These findings indicate that, although the specific targets may vary depending upon cellular context, eHsp90 has the general capacity to increase stem-like properties within PCa cell models.

### Secreted Hsp90 increases prostasphere formation

**Figure 2:**
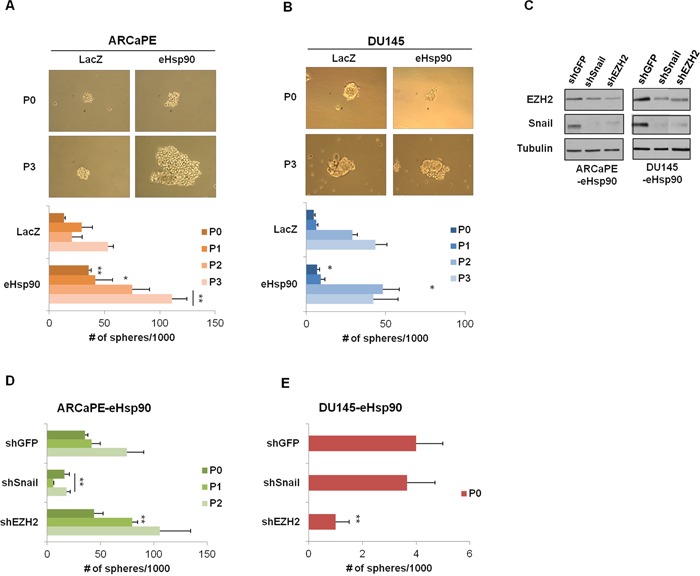
Secreted Hsp90 increases prostasphere formation **A, B**. Representative images of prostaspheres generated at P0 (first generation) and P3 (fourth generation) in ARCaPE-LacZ and ARCaPE-eHsp90 (A) and DU145-LacZ and DU145-eHsp90 (B). Bottom panels depict a graphical representation of the spheres generated from P0-P3 when passaged at 2000 cells per well in a 6 well low-attachment plate. The experimental eHsp90-expressing cells were compared to LacZ controls for each respective passage number. **C**. Immunoblot analysis depicting Snail and EZH2 expression in shRNA transduced ARCaPE-eHsp90 and DU145-eHsp90 cell models in relation to the shGFP control vector. Tubulin is used as a loading control. **D, E**. Graphical representation of the spheres generated from P0-P2 in ARCaPE-eHsp90 (D) and P0 in DU145-eHsp90 (E) following suppression of either Snail or EZH2. Successive generations in DU145-eHsp90 did not produce spheres. The shSnail and shEZH2 experimentals were statistically compared against shGFP for each given passage number. All statistics were performed using the Student's t-test. * = p<0.05, ** p<0.01.

Self-renewing stem cells have increased potential for forming prostaspheres, and Huang et al. have shown that prostaspheres can form fully differentiated prostate basal and luminal cells *in vivo* [48], supporting use of this assay for CSC enrichment. Use of this assay revealed the ability of eHsp90 to increase prostasphere formation in ARCaPE at each passage (from P0-P3), with significance achieved at all generations except P2 (Figure 2A). Moreover, the number of spheres generated consistently increased from P0 to P3 generations. Interestingly, eHsp90 had a less pronounced effect in the DU145 model (Figure 2B). Although there was an increase in sphere generation from P1 to passages P2 and P3, this increase was also noted in the matched LacZ control cells. Therefore, relative to control cells, eHsp90 was only able to elicit a statistically significant increase in DU145 prostasphere formation at the P2 stage, indicating that eHsp90 has a modest effect on prostasphere growth in this cell line.

We previously demonstrated that eHsp90 induced the expression of Snail and EZH2 in ARCaPE [40, 45], and similarly modulated EZH2 expression in DU145 [45]. Given the well documented roles of Snail and EZH2 as modulators of cancer stemness [49–52], we evaluated their potential role in eHsp90-mediated cellular plasticity. As shown (Figure 2C), stable transduction of the indicated models with shRNAs against Snail or EZH2 resulted in a significant decrease in the protein expression of these targets. Interestingly, knockdown of Snail also impacted EZH2 expression, an effect that was reciprocally observed with EZH2 knockdown, and in keeping with a prior report [53]. As shown (Figure 2D), loss of Snail protein impaired sphere formation, an effect which was sustained through multiple generations. Surprisingly, loss of EZH2 in ARCaPE-eHsp90 did not impair prostasphere growth, and in fact, may have enhanced growth at P1. In contrast, loss of Snail in DU145-eHsp90 had no effect upon prostasphere growth, while loss of EZH2 profoundly affected spheroid growth (Figure 2E). These data indicate that the key effectors driving prostasphere growth demonstrate cell-context dependent effects.

### Extracellular Hsp90 enhances ALDH activity via Snail and EZH2

**Figure 3:**
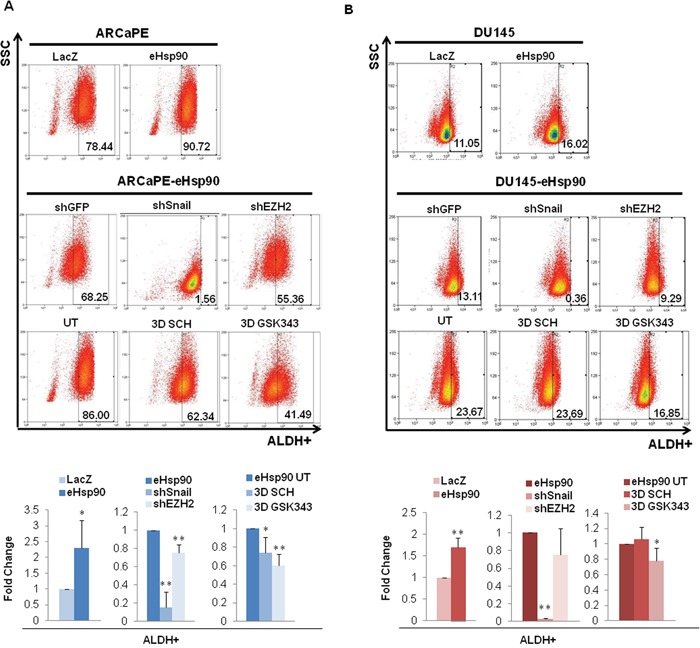
eHsp90 enhances ALDH activity via Snail and EZH2 **A, B**. Top panels show representative flow cytometry scatter plots for the ALDEFLUOR assay of ARCaPE-LacZ relative to ARCaPE-eHsp90 (A) and DU145-LacZ relative to DU145-eHsp90 (B). Middle panels show representative scatter plots for ARCaPE-eHsp90 and DU145-eHsp90 transduced with either an shGFP vector control, shSnail, or shEZH2. Lower panels show representative scatter plots for ARCaPE-eHsp90 and DU145-eHsp90 treated with 100 nM SCH or 500 nM GSK343 over a 3 day period. Comparisons included eHsp90-expressing cells relative to the matched LacZ control. As indicated, additional treatments or cell derivatives were compared relative to the matched untreated eHsp90-expressing cells. Combined analysis of replicate assays are shown in accompanying graphs. All statistics were performed using the Student's t-test. * = p<0.05, ** p<0.01.

To determine whether eHsp90 impacted additional properties associated with stem cell behavior, we next assessed the role of eHsp90 signaling on ALDH1A1 activity. The Aldefluor assay isolates the ALDH^+^ population, which is highly enriched in tumor initiating cells (TICs) in prostate and other progenitors [54], and ALDH1 is commonly used as a marker to predict clinical outcome in prostate cancer patients [55]. Of note, some of the previously noted downstream effectors of eHsp90 signaling, such as Snail and EZH2, have both been linked to ALDH1A1 expression and/or activity [50, 56, 57]. Utilizing the established ALDEFLUOR assay, we observed that eHsp90 significantly enhanced ALDH1A1 activity between 1.5-2 fold in both ARCaPE and DU145 (Figures 3A, 3B, uppermost panels, and respective bottom left graphs). Interestingly, loss of Snail more dramatically attenuated eHsp90-dependent ALDH1A1 activity in both ARCaPE and DU145 relative to loss of EZH2, the latter of which had a very modest, but significant effect in ARCaPE (Figures 3A, 3B, middle panels and bottom middle graphs, experimental controls shown in Supplementary Figure 1A). These data support previously published findings indicating a role for Snail regulation of ALDH1A1 activity [58, 59].

Both Snail and EZH2 may be influenced by ERK signalling [45, 60, 61], and ERK signaling has been further implicated in regulating prostate cancer stem cells [62]. Given this link, along with our previous demonstration that ERK is a critical signal integrator for eHsp90 action [45], we assessed whether ERK targeting impacted the eHsp90-mediated increase in ALDH1A1 activity. Surprisingly, targeting ERK with the specific small molecule SCH-229874 (referred to as SCH) had a very modest effect in ARCaPE-eHsp90, and essentially no effect in DU145-eHsp90 (Figures 3A, B, lower panels, and right-most graphs). We further investigated the effects of EZH2 targeting, as the pharmacologic blockade of EZH2 should be more effective relative to RNAi suppression. EZH2 targeting with the small molecule inhibitor GSK343 resulted in nearly identical results to those produced by shRNA-dependent targeting, thereby validating a modest, and possibly cell-context dependent role for EZH2 in modulation of ALDH activity. Collectively, our findings also suggest that ERK may not represent a central signaling portal for eHsp90 function within the context of ALDH activity. Alternatively, Snail appears to play a dominant role in an ERK-independent manner.

### Extracellular Hsp90 increases the side population in part through an EZH2 dependent pathway

**Figure 4:**
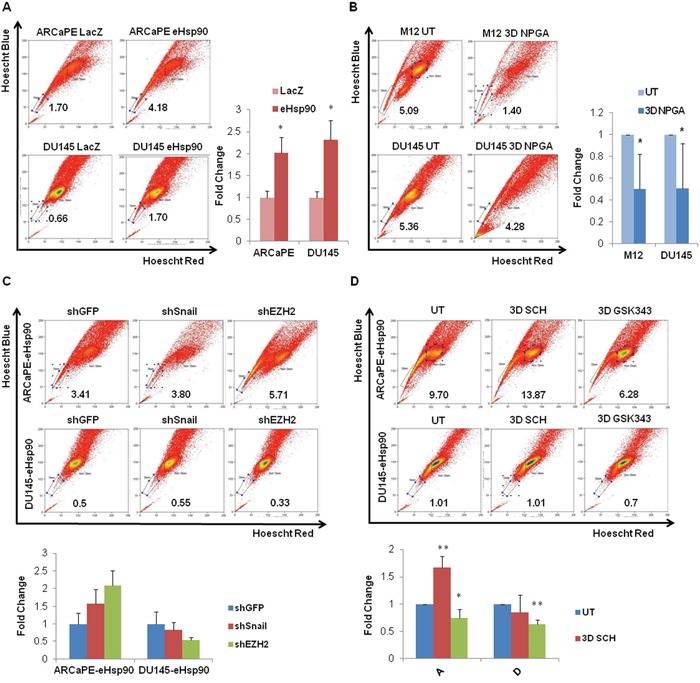
eHsp90 increases the side population in part through an EZH2 dependent pathway **A**. Representative flow cytometry scatter plots for the side population of ARCaPE-LacZ relative to ARCaPE-eHsp90 and DU145-LacZ relative to DU145-eHsp90 are shown, along with graphs demonstrating the results of the combined analysis of replicate assays. **B**. Representative flow cytometry scatter plots for the side population of M12 and DU145, each treated with 1 uM NPGA for 3 days. Comparisons included eHsp90-expressing cells relative to the matched LacZ control (A) or drug treated relative to untreated control cells (B). Shown are quantified results of the combined analysis of replicate assays. **C**. Representative flow cytometry scatter plots for the side population of ARCaPE-eHsp90 and DU145-eHsp90, each stably transduced with plasmids encoding shGFP (vector control), shSnail or shEZH2 are shown, along with graphs demonstrating the results of the combined analysis of replicate assays. **D**. Representative flow cytometry scatter plots for the side population of ARCaPE-eHsp90 and DU145-eHsp90 treated with 100 nM SCH or 500 nM GSK343 over a 3 day period are shown, along with graphs demonstrating the results of the combined analysis of replicate assays. Comparisons included eHsp90-expressing cells relative to the matched LacZ control. As indicated, eHsp90-expressing shRNA cell derivatives were compared relative to matched shGFP transduced cells (C), while eHsp90-expressing drug treated cells were compared to matched untreated controls (D). All statistics were performed using the Student's t-test. * = p<0.05, ** p<0.01.

Cancer stem-like cells demonstrate an enhanced ability to efflux toxins and drugs, a property that contributes to their drug resistant phenotype. These cell types can be detected by the ‘side population’ assay, as assessed by the extent of dye efflux [43, 63]. As shown (Figure 4A), eHsp90 increased the dye-effluxed side population by approximately 2-fold in eHsp90-expressing ARCaPE and DU145 models relative to their respective LacZ controls (experimental controls shown in Supp Figure 2A). To further validate a functional role for eHsp90, we determined whether the eHsp90 inhibitor NPGA would reverse these trends. We therefore evaluated the effects of NPGA in both DU145 and M12, the latter an aggressive mesenchymal cell line [64] that depends upon sustained eHsp90 signaling for a subset of its aggressive traits [40, 45]. As shown (Figure 4B), NPGA treatment significantly reduced the side population in both ARCaPE-eHsp90 and DU145-eHsp90 models relative to their respective LacZ controls.

We next evaluated the relative involvement of Snail, EZH2 and ERK in regulation of the eHsp90-mediated side population. As shown (Figure 4C, experimental controls in Supplementary Figure 2B), the genetic suppression of Snail or EZH2 failed to negatively impact the percentage of cells characterized as belonging to the side population. We next utilized pharmacologic inhibitors of ERK and EZH2 to further validate their potential role in regulating this cell population. Whereas genetic suppression of EZH2 did not demonstrate a significant effect, GSK343 treatment did elicit a modest, but statistically significant reduction in the side population in ARCaPE-eHsp90, and exhibited a more robust effect in DU145-eHsp90 (Figure 4D). Interestingly, ERK targeting had no negative impact upon the side population. These results indicate that pharmacologic targeting of EZH2 more effectively attenuated EZH2 activity relative to genetic suppression, and that EZH2 plays a contributory role in promoting eHsp90-dependent expansion of the side population.

### Blockade of eHsp90 signaling impairs spheroidogenesis

**Figure 5:**
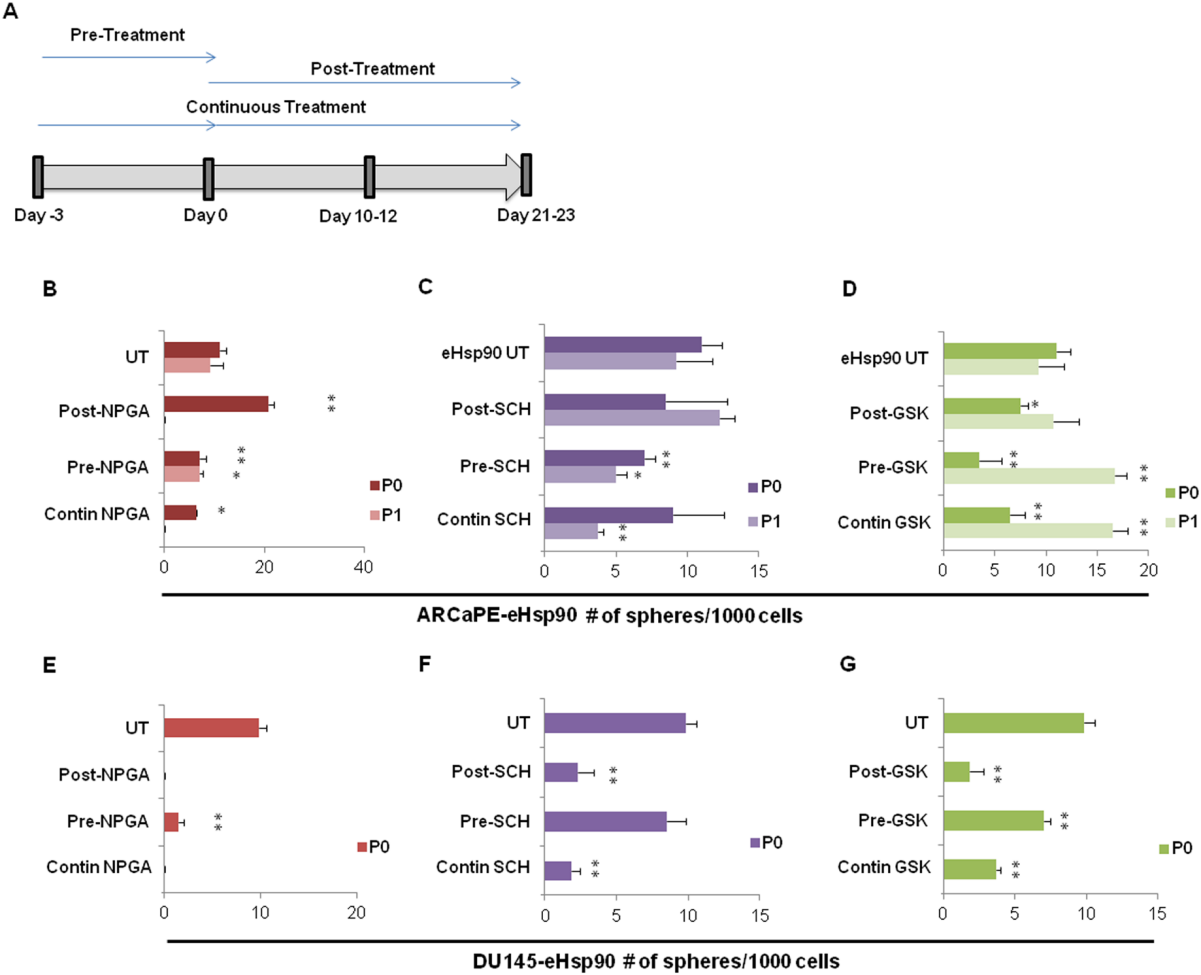
Blockade of eHsp90 signaling impairs spheroidogenesis **A**. Schematic representation of treatment regimens followed for cells treated with either 1 uM NPGA, 100 nM SCH or 500 nM GSK343. Cells were separated into 3 treatment groups, and each group was placed into spheroid suspension at Day 0. The pre-treatment group was subjected to the indicated inhibitors for 3 days prior to the spheroid suspension assay. The post-treatment group was treated with the indicated inhibitors upon initial suspension into the spheroid assay. The continuous treatment group (Contin) was pre-treated for 3 days with the indicated inhibitors, and subsequently maintained under the specified conditions following initiation of spheroid culture. ARCaPE-eHsp90 **B-D**. and DU145-eHsp90 **E-G**. were subjected to the indicated inhibitors and evaluated under the 3 experimental conditions as outlined in A. The number of spheres generated by each treatment status was counted for each generation and is graphically depicted. The sphere treatments were all compared against the untreated (UT) control sample for each given passage. All statistics were performed using the Student's t-test for the treated versus the untreated control. * = p<0.05, ** p<0.01.

Our collective results support a role for eHsp90 as a facilitator of prostasphere growth. We next explored the dynamics of eHsp90, ERK and EZH2 action with respect to their requirement for spheroid growth. The general treatment schema is depicted in Figure 5A, wherein treatments were evaluated either prior to initiation of suspension conditions (Pre-treatment), targeting cells post suspension (Post-treatment), or continuous exposure of cells both prior to and immediately following initiation of spheroid seeding (Contin-treatment). This treatment plan revealed that targeting eHsp90 in ARCaPE-eHsp90 prior to spheroid conditions, or under continuous conditions, reduced prostasphere growth at passage P0, with the latter condition completely preventing spheroid growth at passage P1. Although NPGA appeared to increase sphere number post treatment, the spheres were dramatically smaller relative to untreated spheres (not shown). Moroever, under Post-NPGA treatment conditions, no spheres were recovered from the P0 to P1 passage. These data support the notion that eHsp90 action is continuously required for robust prostasphere growth.

A similar experimental strategy was utilized to evaluate the requirement for ERK and EZH2 activity upon ARCaPE-eHsp90 prostasphere growth. ERK targeting by SCH impaired ARCaPE-eHsp90 prostasphere growth when administered prior to suspension conditions, consistent with its ability to attenuate prostasphere growth under continuous conditions (Figure 5C). Given that Post-SCH treatment was ineffective, our results indicate that ERK signaling is more critical prior to sphere generation. The effects of EZH2 targeting were unexpected. While EZH2 targeting reduced prostasphere growth under both pre- and post-treatment conditions, as well as under continuous treatment, this effect was only observed for passage P0 (Figure 5D). Upon transition from P0 to P1, ARCaPE-eHsp90 appeared relatively resistant to EZH2 targeting, and in fact, an increase in sphere growth was noted. Although the precise mechanism for this effect is not clear, this result may be due to a compensatory feedback mechanism or emergence of a more resistant CSC population.

We next evaluated the involvement of eHsp90, ERK and EZH2 in DU145-eHsp90 prostasphere growth. Consistent with our results with ARCaPE-eHsp90, eHsp90 targeting with NPGA dramatically reduced prostasphere growth in all treatment regimens, thereby validating its role as a critical mediator of sphere growth and regeneration. Interestingly, ERK targeting in DU145 was most efficacious when administered after initiation of spheroid growth (Figure 5F), in contrast to its impact in ARCaPE-eHsp90 prior to spheroid generation. EZH2 was important for DU145-eHsp90 prostasphere growth, demonstrated by its ability to attenuate spheroid growth in all treatment regimens (Figure 5G). As we were unable to passage DU145 under these conditions beyond passage P0, we did not observe a compensatory effect upon EZH2 targeting, as was noted in ARCaPE-eHsp90. Our data demonstrate that eHsp90, ERK and EZH2 are important effectors of prostasphere growth, but that they may exert differential effects upon sphere initiation relative to successive propagation.

### Increased stem marker expression in tumor cells with elevated surface-bound Hsp90

**Figure 6:**
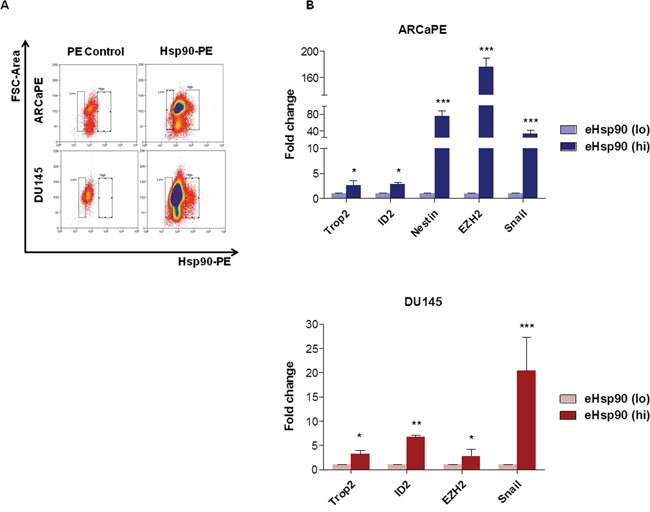
Increased stem marker expression in tumor cells with elevated surface-bound Hsp90 **A**. Representative flow cytometry scatter plots for surface-bound Hsp90-alpha in ARCaPE and DU145. Samples treated with secondary Rabbit IgG-PE were used as a negative control (denoted PE Control). Surface-bound Hsp90-PE was defined as the area of events with higher PE staining not found in the negative control population, which ranged from 2-5% for each cell line. **B**. RNA was harvested from each of these respective cell populations and qRT-PCR was performed for each of the indicated stem-like gene targets. All statistics were performed using the Student's t-test for the treated versus the untreated control. * = p<0.05, ** = p<0.01, *** = p<0.001.

Our data indicated that eHsp90 modulated several facets associated with stem-like cell behavior. Given that eHsp90 may be found in both secreted and cell surface-bound forms, we next evaluated whether cells exhibiting elevated surface-bound Hsp90 may enrich for a stem-like cell population. As shown, incubation of nonpermeabilized ARCaPE or DU145 cells with labeled anti-Hsp90 alpha antibody enabled isolation of respective populations with high and low surface Hsp90 expression (Figure 6A). Remarkably, the stem markers Trop2 and ID2 were significantly elevated in both ARCaPE and DU145 cell populations exhibiting high surface Hsp90 expression, while Nestin was upregulated in eHsp90-isolated ARCaPE (Figure 6B). Interestingly, EZH2 and Snail were also upregulated in Hsp90 surface bound cell populations from both ARCaPE and DU145. These findings indicate that prostate cancer cells marked with surface Hsp90 enriches for a distinct cell population that possess elevated expression of a cohort of stem-like gene targets.

### Surface-bound Hsp90 co-segregates with a subset of PSA(lo)-expressing cells

The role of stem-like cancer cells in the development of prostate cancer is not well characterized. Although prostate cancer is considered an androgen-driven pathology, particularly in the early stages, a number of studies have reported the existence of a cell population with diminished AR activity, and corresponsibly reduced prostate-specific antigen (PSA) expression [21, 65]. It has been shown that this PSA(lo) cell population segregates with cells exhibiting CSC features. Given our data that surface Hsp90 may ‘mark’ a population of stem-like prostate cancer cells (Figure 6), we next evaluated whether surface Hsp90 correlated with AR expression. The well-characterized human prostate cancer line LNCaP, as well as the mouse line Myc-CaP [66] were utilized as androgen-dependent cell models. To facilitate detection of AR activity, these cell models were transduced with plasmids encoding a PSA-GFP vector, whereby GFP expression is driven by PSA activity [21]. This approach allows for the isolation of GFP-expressing cell types as a functional surrogate for AR activity.

**Figure 7:**
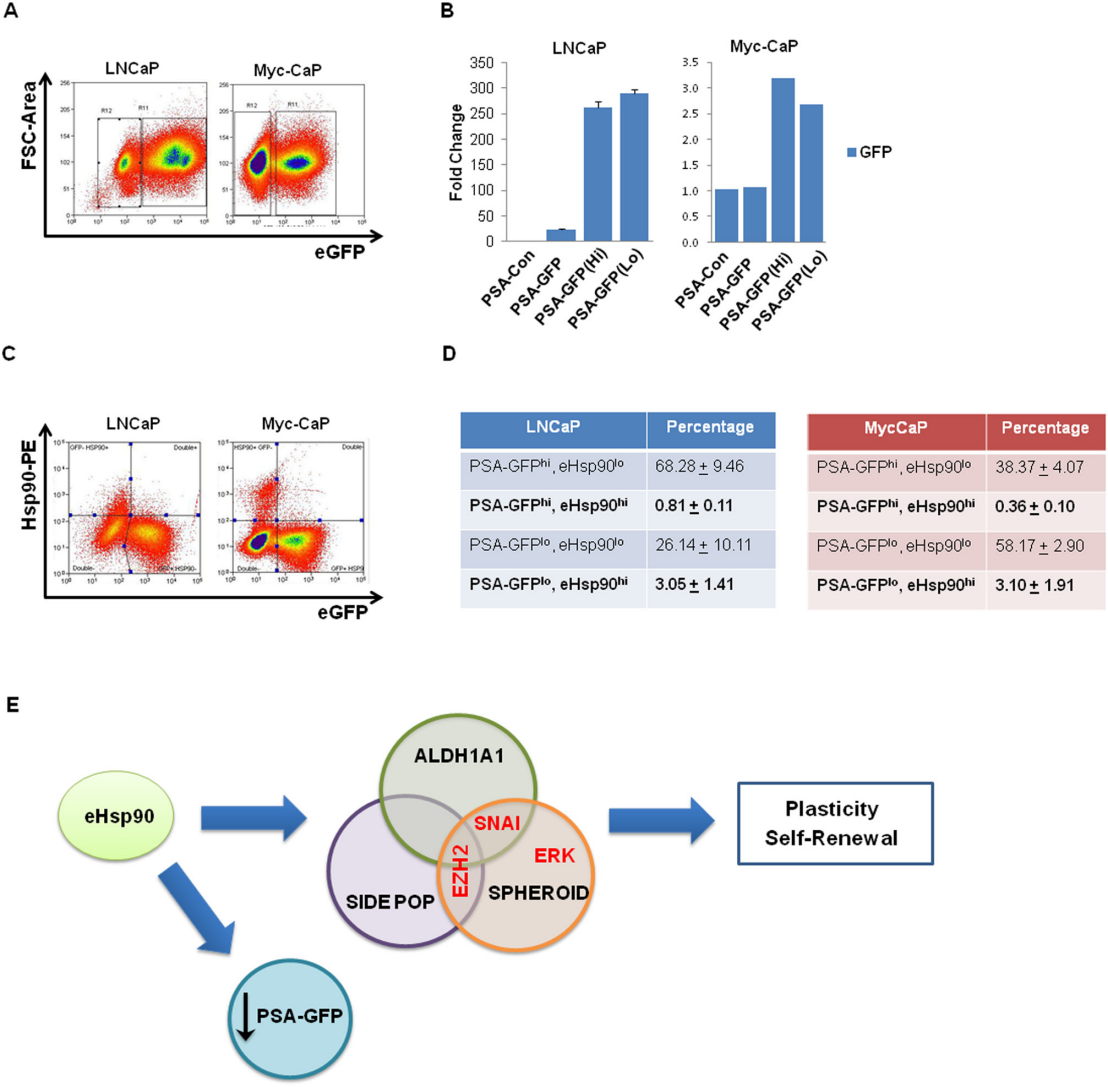
Surface-bound Hsp90 co-segregates with a subset of PSA(lo)-expressing cells **A**. Androgen receptor (AR) responsive LNCaP and Myc-CaP prostate cancer cells were transiently transduced with lentiviral particles encoding PSA-GFP and GFP expression was assessed by flow cytometry. Approximately 70-80% of LNCaP are GFP+, while approximately 40% of MycCaP demonstrate similar positivity. PSA-Con refers to nontransduced control cells, while PSA-GFP refers to non-sorted bulk cells. **B**. Quantitative PCR analysis was performed on the indicated cell populations to determine the relative DNA expression of GFP, indicative of transduction efficiency. The populations are identified as follows: untransduced (PSA-con), transduced unsorted (PSA-GFP), and flow sorted PSA-GFP positive and negative (PSA-GFP(Hi)) and PSA-GFP(Lo)). **C**. Representative flow cytometry scatter plots demonstrating the relation between surface-bound Hsp90-alpha and PSA-GFP expression in LNCaP and MycCaP cells. **D**. Tabular depiction of replicate flow cytometry analyses for surface-bound Hsp90 in LNCaP and Myc-CaP cells. **E**. Model depicting the role of eHsp90 in supporting stem-like cellular heterogeneity. Collectively, our data support a model whereby secreted Hsp90 increases stem-like properties in prostate tumor cells. This was demonstrated by increased prostasphere growth, and expansion of both the side population (indicative of dye efflux and drug transporter activity) and ALDH activity. As indicated, the effectors EZH2 and Snail appear to differently impact seveal of these metrics. In tandem, tumor cells with elevated surface Hsp90 appear to correlate with lower levels of PSA-regulated GFP, indicating a potential inverse correlation between surface Hsp90 and androgen receptor (AR) regulation.

We first confirmed detection of intact cell populations exhibiting high and low GFP expression (Figure 7A). We next confirmed that the respective PSA(lo) populations were due to diminished AR activity, rather than the result of inefficient transduction. Although the overall activity of AR was higher in LNCaP, we detected similar levels of GFP gene integration within the sorted PSA(hi) and PSA(lo) populations (Figure 7B), indicating that differences in PSA expression within these populations were most likely reflective of AR activity, rather than to differences in GFP gene integration and respective gene copy. Having validated the utility of the system, we next evaluated PSA expression within the context of surface Hsp90. Representative flow cytometry scatter plots demonstrating both surface bound Hsp90 and GFP expressing populations in these cell models is shown (Figure 7C), along with the corresponding tabular depiction these quantified data (Figure 7D). The distribution of the 4 relevant cell populations reveals that, although elevated AR activity is observed in both eHsp90 high and low populations, AR activity preferentially trends with eHsp90^lo^ cells. This trend is corroborated in that the eHsp90(hi) cell populations (in bold) preferentially segregate with a subset of the CSC-associated PSA(lo) cell population (Figure 7D). These data highlight an inverse trend in that high surface Hsp90 expression tends to ‘mark’ a subpopulation of PCa cells with diminished PSA-GFP expression.

## DISCUSSION

It is well established that CSCs contribute to cancer cell plasticity and confound therapeutic approaches [67]. However, the effective targeting of CSCs has posed a significant challenge, due in part to the multitude of cellular stimuli capable of supporting and expanding this cell population [68]. To our knowledge, this study represents the first report to identify eHsp90 as a regulator of prostate CSCs. This premise is supported by integrated data obtained from multiple well-established assays designed to evaluate CSC-like properties. Using isogenic lines to establish a role for Hsp90 secretion, we determined that eHsp90 promotes the existence of cell populations with properties of enhanced self-renewal, increased efficiency of prostasphere formation, increased side population and increased ALDH activity. Moreover, eHsp90 increased the expression of several stem-like gene targets. Although the eHsp90-mediated induction of these gene targets exhibited variability within the evaluated PCa lines, Trop2 (tumor-associated calcium signal transducer 2) was induced in both instances. Trop2 has been reported as a marker for prostate stem cells [69], and its expression is associated with sphere formation, self-renewal and tissue regeneration [70, 71]. Also of note, the developmental transcription factor SOX4, upregulated in DU145, has been shown to possess oncogenic activity in prostate cancer [72, 73], wheres Nestin has been associated with the drug resistant prostate stem population [74]. Collectively, our data demonstrate that eHsp90 broadly supports the development of stem-like cell populations within the context of PCa.

The EMT developmental process is a critical regulator of cell fate decisions. Given our prior findings linking eHsp90 and EMT [40, 45], we evaluated the potential involvement of the eHsp90 effectors EZH2 and ERK within the context of eHsp90-mediated stemness. We also assessed the role of Snail, given its identity as an eHsp90 target [40], and as a well-established effector of both EMT activation and cancer stemness [75–78]. Interestingly, although our findings implicate ERK, EZH2 and Snail as participants in eHsp90-driven stem-like traits, our data demonstrate that these effectors modulate different attributes of stem-like behavior. As summarized schematically (Figure 7E), while blockade of EZH2, Snail and ERK impacted eHsp90-directed prostasphere growth, EZH2 regulated both the side population and cells marked by ALDH activity, while Snail preferentially affected the ALDH population. These data indicate that although Snail and EZH2 may co-regulate similar stem-like populations, consistent their demonstrated ability to functionally cooperate in regulating EMT events and gene targets [79, 80], they may also influence distinct cell types, indicating a divergence of function. Functional crosstalk has also been reported for ERK and EZH2 [45, 60, 81], and between ERK and Snail [82, 83]. However, as is evident from our findings, ERK was unable to modulate either the side population or the ALDH population, indicating that ERK does not significantly collaborate with either EZH2 or Snail within this context. Nonetheless, our data demonstrating a critical role for Snail in prostasphere growth is consistent with other studies utilizing PCa models [49].

It is well known that cellular heterogeneity is a major component of PCa development and maintenance [84], a feature that complicates both the diagnosis and treatment of cancer. Multiple subsets of stem-like tumor initiating cells (TICs) are suspected of supporting this tumor heterogeneity [85, 86]. Our collective findings support the existence of a heterogeneous stem-like cell population, which is further validated by our data demonstrating the ability of multiple effectors to govern overlapping, and distinct, stem-like cell populations. While it is presently unclear how stem markers and their respective functional properties relate within a clinical setting, it is likely that multiple stem-like populations cooperate to drive cancer at distinct stages. Reports have validated the existence of stem cell markers found in bone metastatic cancer, although their relation to EMT effectors was inconsistent [87]. Likewise, global analysis of CSCs compiled from numerous studies has demonstrated variable EMT profiles in prostate CSCs [88], further attesting to tumor and CSC heterogeneity. Additional studies are warranted to better understand whether eHsp90-regulated CSC populations harbor properties associated with TICs, metastatic potential, and/or therapeutic resistance.

Extracellular Hsp90 also may be localized in a cell surface-bound form, as recently reviewed [33]. Although it is unclear whether soluble or surface forms represent separate or dynamic populations [89], both forms are preferentially detected in diverse tumor types. We therefore evaluated whether PCa tumor cells ‘marked’ with surface Hsp90 may represent a unique cell population. Intriguingly, tumor cells with elevated surface Hsp90 expression (eHsp90^hi^) isolated from ARCaPE and DU145 demonstrated a profound increase in a cohort of stem-like effectors, such as Trop2, ID2 and Snail. While many of these expression trends were similarly observed within the nonselected eHsp90-expressing bulk cells, isolation of PCa cells with surface Hsp90 exhibited a dramatic increase, indicating that surface eHsp90 may indeed mark a distinct CSC population. In further support of this premise, expression levels of Snail message were substantially increased in this population, indicating that these cells may be reliant upon Snail action. Using a similar sorting strategy to evaluate surface eHsp90 in androgen dependent models, we show that eHsp90(hi) cells preferentially segregate with PSA(lo) cells. This PSA(lo) subpopulation has been shown to harbor self-renewing CSCs that are refractory to castration, correlated with a drug-resistant mesenchymal phenotype [65], and are present in patient populations [21, 22]. It remains to be determined whether surface eHsp90 supports the development of, or is required for, maintenance of this population. Corroborating our findings, a recent report demonstrated that surface eHsp90 correlates with stem-like markers in breast cancer [90]. Moreover, eHsp90 was shown to be required for breast cancer clonogenic potential, indicating that surface localization of eHsp90 in tumors may be a conserved property of aggressive subtypes.

The discovery that eHsp90 may be correlated with a stem-like tumor population has broad implications. Numerous environmental stressors have been reported to increase both secreted and surface bound expression of eHsp90. Given this correlation, it is possible that within a clinical setting, therapeutic modalities such as chemotherapy and radiotherapy would stimulate eHsp90 expression, and in turn support the survival and maintenance of a CSC population, a result predicted to contribute to the enriched stem-like population observed following therapy. Hsp90 inhibitors are generally well known to sensitize the drug-resistant side population of tumor cells [91–93] supporting a linkage between Hsp90 and cancer stemness. As these drugs are predicted to nonspecifically target both intracellular and extracellular populations of Hsp90, it remains an open question whether more specific targeting of eHsp90 may elicit a more direct effect upon the CSC population. A recent report supports such a directed strategy in that therapeutic targeting of eHsp90 synergized with conventional therapeutics [90]. Our findings also raise the question of whether eHsp90 may broadly contribute to the de-differentiation of cell types to facilitate the generation of stem-like cell populations. Although such dynamic interconversions have been demonstrated within the context of malignancy [94], a similar mechanism may be relevant for wounding and repair. Cellular plasticity is essential for tissue remodeling, and eHsp90 has been shown to support tissue repair [95]. Taken together, it is plausible that cancer cells have hijacked an expanded and conserved function of eHsp90, thereby enabling tumor cells to acquire the enhanced plasticity required for survival upon exposure to the environmental stress associated with malignancy and therapy.

## MATERIALS AND METHODS

### Cell models and culture

ARCaPE cells were purchased from Novicure Biotechnology, DU145, LNCaP, and MyC-CaP were purchased from ATCC, M12 was obtained from Joy Ware (VCU Medical Center). 293T cells for lentiviral transductions were obtained from Scott Eblen (MUSC). The ARCaP cell pair was cultured in T-media (Invitrogen) supplemented with 5% heat-inactivated fetal bovine serum (FBS from Gibco 10437-028) and 1% penicillin/streptomycin (Pen/Strep from Hyclone SV30010). The M12 was cultured in T-media with 0.5% heat-inactivated FBS with 1% Pen/Strep. LNCaP and DU145 were maintained in RPMI-1640 (Hyclone SH30096.02) supplemented with 10% FBS with 1% HEPES, 1% sodium pyruvate, 1% glutamine, and 1% Pen/Strep. The 293T and myc-Cap cells were cultured in DMEM (Hyclone SH30285.02) with 10% FBS. Cells cultured in the spheroid assay were all cultured in 6 well ultra-low attachment plates in DMEM/F12 with 1X N2 (Gibco 17502-048) and 1x B27 (Gibco 17504-044) as previously described [18].

### Reagents and antibodies

Recombinant Hsp90a protein was purchased from Enzo Life Sciences (ADI-SPP-776). DMAG-*N*-oxide modified geldanamycin (NPGA) was synthesized by Chris Lindsey and Craig Beeson (Drug Discovery, Medical University of South Carolina). ERK1/2 Inhibitor, SCH-229874 (S7101, Lot# 3), and EZH2 inhibitor, GSK343 (S7184), were purchased from SelleckChem. Verapamil was purchased from Tocris (0654) and Hoescht 33342 was purchased from Invitrogen (H3569). Antibodies for Snail (3895) and EZH2 (5246) were purchased from Cell Signaling. Antibody to a-tubulin (T6074) was purchased from Sigma and V5 antibody (NB600-381) was purchased from Novus Biologicals. Antibodies for Hsp90-alpha (ADI-SPA-840) and Hsp90-alpha-PE conjugate (ADI-SPA-840PE-200, Lot# 03051244C) were obtained from ENZO Lifesciences.

### Viral constructs, transfections, and transductions

Plasmids for transfection were processed and purified utilizing QIAGEN's Maxi-Prep Hi-Speed kit per manufacturer's instructions. The pLenti6.3-V5-LacZ and eHsp90 plasmids were generated and purchased from Genecopoeia as described [40]. The shEZH2 plasmid was purchased from the MUSC shRNA Technology Core (Sigma Mission Library cat# SHCLND-NM_004456) and the shSnail plasmid was obtained from Gregory Longmore (Washington University, St. Louis, MO). The PSA-GFP vector was obtained from Dean Tang (MD Anderson, Houston, TX). All transfections for production of lentiviruses were performed as previously described [40, 45] in 293T cells. Briefly, 293T cells were cultured in 10 cm plates in antibiotic-free media for a full 24 hours prior to transfection. On the day of transfection, a 6:1 ratio volume of Fugene (Promega E2691) to DNA amount was added to 800 uL of serum-free DMEM and incubated for 15 minutes at room temperature. The plasmid DNA of interest and two packaging co-plasmids (VSV-G and DR891) were added to the Fugene-media mix and incubated an additional 15-30 minutes at room temperature. The mixture was then added to the 10 mL of media on the 293T cells. At 24 and 48 hours post-transfection media was collected and ultra-centrifuged at 50,000x g to pellet viral particles. The supernatant was removed and particles were resuspended into 1 mL of serum-free RPMI or T-media. Viral particles were either used directly or aliquoted and stored at -80C.

All lentiviral transductions were performed similarly. Cells to be infected were seeded into either a 12 or 24 well plate with 0.5 to 0.75 mL culture media and cultured to 40-50% confluency. Fresh media was added directly prior to transduction containing 8 ug/mL Polybrene. Concentrated viral particles were added at 50-150 uL to designated wells. A second round of transduction was performed in the same manner 24 hours after the first transduction. After 24-48 hours from the second transduction, cells were trypsinized and plated into 10 cm dishes and cultured for an additional 48 hours. At 48 hours post-plating the media was changed to selection media containing either puromycin or blasticidin depending on plasmid resistance. Surviving cells were then cultured for characterization and further experiments.

### Western blotting

Western blots were performed as previously described [40, 45]. Briefly, protein lysates from cells were collected upon lysis in RIPA-lysis buffer and centrifuged to pellet cellular debris. Media from serum-starved cultures were collected and concentrated through a Millipore Amicon Ultra 3K column and analyzed for protein concentration. 20-50 ug of total cell lysate proteins were loaded onto a 10% SDS-PAGE gel and allowed to migrate through the gel at 90-100V. The resultant gels were then transferred onto a nitrocellulose membrane overnight, followed by blocking in 10% milk. Primary antibodies in 5% milk were added for overnight incubation at 4°C. Membranes were subsequently washed in TBST and secondary antibodies conjugated to HRP were added in 5% milk and incubated for a minimum of one hour at room temperature. Membranes were subsequently washed and developed with chemiluminescent regents.

### Spheroid and self-renewal assays

To analyze prostasphere formation, adherent cells were trypsinized, counted, and re-suspended to 1 x10^5^ cells per mL. Cells were seeded at 2000 cells per well in a 6 well low-attachment plate to inhibit plate attachment. Spheroid media containing DMEM/F12, B27, and N2 was added at 2 mL per well. 1 mL of media was added every 3-4 days and spheres were counted on Day 10-14 for each passage. Criteria for spheroid growth constituted a minimum of 5 cells adhered together. Images were captured with an inverted Nikon Eclipse TE 2000-S microscope with 10× magnification. Spheres were passaged by collecting the spheres post-imaging and centrifuging in a 15 mL conical. Spheres were then re-suspended in 100-250 uL Accutase (Corning, 25-058-CI) cell detachment solution. They were then incubated at 37°C for a minimum of 10 minutes and a maximum of 20 minutes for dissociation of the cells. Cells were then counted and re-seeded to new 6 well low attachment plates with spheroid media. Spheres were passaged for no more than five generations. Resultant sphere numbers for each passage was graphed in a bar graph as number of spheres per 1000 cells. Statistical differences between cell lines or treatments were determined via one-way ANOVA followed by Student's t-test for each individual passage.

Self-renewal assay was performed as previously described [96]. Briefly single cells were suspended at 1000 cells per mL following the first or second generation of spheroid formation. 2 uL of cells were seeded into a 96 well low-attachment round bottom plate with 100 uL of spheroid media and verified for single cell dispersal 24 hours post seeding. The number of wells with a single cell provided the base number to assess renewal. 50 uL of fresh spheroid media was added to each well every 3-4 days and the number of spheres formed from the initial wells was counted at 10-14 days post-seeding. The percent efficiency of sphere formation was determined by dividing the number of spheres formed over the initial number of wells with a single cell multiplied by 100. Self-renewal efficiency was calculated by dividing the efficiency of sphere formation at P2 over the efficiency of sphere formation at P1 multiplied by 100.

### Quantitative RT-PCR

RNA purification from cells was performed following a TRIzol/chloroform extraction procedure according to the manufacturer's recommendations (Qiagen miRNeasy kit #217004). Quantitative RT-PCR was performed utilizing the two-step process involving converting the mRNA to complementary DNA (cDNA) followed by real-time PCR. Isolated mRNA was converted into cDNA (Bio-Rad iScript cDNA synthesis kit) and amplified for quantitative PCR reactions. Primers were purchased from Integrated DNA Technologies and the sequences are listed in Table 1. All quantitative real-time PCR reactions were performed in technical triplicates from at least two biological replicates. The data shown are presented as mean ± S.D. with differences in treatment groups defined as statistically significant at p < α = 0.05, as calculated from Student's t-test.

**Table 1:** Primers used for RT-PCR

Name	Accession #		Sequence
Trop2	NM_002353	SenseAntisense	CCCTTTCGGTCCAACAACAGGAAAAACGATCCCGGGTTGTCATACAGA
Sox4	NM_003107	SenseAntisense	CTCCAGCCTGGGAACTATAAGGAGGTGGGTAAAGAGAGAA
ID2	NM_002166	SenseAntisense	TCCCAGGGTGTTCTCTTACTTGGAAAACCTTCCAACTGCAGAAAGGGC
GAPDH	NM_002046	SenseAntisense	TCGACAGTCAGCCGCATCTTCTTTACCAAATCCGTTGACTCCGACCTT
Nestin	NM_006617	SenseAntisense	AGAGCGTAGAGGCAGTAAAACAGTGGTGCTTGAGTTTC
MDR1	NM_000927	SenseAntisense	ATGCTCTGGCCTTCTGGATGGGAATGGCGATCCTCTGCTTCTGCCCA
ACTB	NM_001101	SenseAntisense	GATCAGCAAGCAGGAGTATGAAGGGTGTAACGCAACTAAG
Snail	NM_005985.3	SenseAntisense	CTCCCTCTTCCTCTCCATACTGGCAGTGAGAAGGATGT
EZH2	NM_004456	SenseAntisense	AGAGGACGGCTTCCCAATAACAGTTTCAGTCCCTGCTTCCCTATCACT

### Flow cytometry assays

All flow cytometry experiments were performed utilizing the Regenerative Medicine Core facility at MUSC. Samples were processed through the MoFlo Astrios and were either sorted into selected populations and/or analyzed for fluorescent emissions. The side population was performed as previously described [97]. In detail, cells were trypsinized, counted, and resuspended at 1 × 10^6^ cells per mL in culture media. 50 uM of the ABC transporter inhibitor, verapamil, was added to labeled microcentrifuge tubes as an inhibitor-negative control for the side population. 1 mL of cells was added to either an empty microcentrifuge tube or an inhibitor labeled tube. Hoescht 33342 nucleic acid stain was added at 5 ug/mL to every tube. All tubes were subsequently protected from light and incubated in a 37C water bath for 2 hours. Cells were then centrifuged and supernatant was removed. Cells were resuspended in 0.5 to 1 mL PBS + 2% FBS and kept on ice. Additional supplements of 10 ug/mL Propidium Iodide and 10 uL of DNase 1 were added to assess cell viability and reduce cell-cell adhesion. Cells were then processed through the flow cytometer utilizing the UV laser to gate for emission spectra of 620 nm on the x-axis (Hoescht Red) and 448 nm (Hoescht Blue) on the y-axis. The gating was performed against the control cells treated with 50 uM verapamil to inhibit dye efflux. The percentage of events indicated within this gated area was labeled as the percentage of side population positive cells. Each experiment was performed a minimum of three times to obtain data suitable for statistical analysis, with analysis performed via Student's t-test. If no side population was present, the experiment was repeated a second time to verify the lack of a side population.

ALDH activity assay was performed utilizing the ALDEFLUOR kit obtained from StemCell Technologies (# 01700), which contains a substrate that becomes fluorescent upon catalysis via ALDH1A1. Cells were trypsinized, counted, and resuspended at 1 x10^6^ cells per mL in ALDEFLUOR Assay Buffer. 5 uL of the provided inhibitor reagent DEAB was added to the labeled tubes. 500 uL of cells were added to the non-inhibitor containing tubes and a subsequent 500 uL of ALDEFLUOR Assay Buffer was added. 5 uL of the ALDEFLUOR Reagent was added to the tubes containing the cells, mixed, and 500 uL of the cell mixture was quickly transferred to the labeled inhibitor tube. All tubes were incubated in a 37C water bath for 15 minutes for ARCaPE cells or 30 minutes for DU145 cells. Cells were transferred to an ice bucket then centrifuged, supernatant removed, and fresh ALDEFLUOR buffer was added at 500 uL per tube. Cells were kept on ice and propidium iodide was added similar to the above assay. Cells were then processed through the flow cytometer and excited using the FL1 laser (488 nm) to generate an emission spectra at 513 nm. Cell events were plotted with the FL1 emission spectra on the x-axis (ALDH+) and the side-scatter on the y-axis. Gating for positively stained cells was determined by analyzing the area of cell events missing from the DEAB treated cells. ALDH+ cells were then gated as cells occupying the corresponding empty space in non-DEAB treated cells. The percentage of events indicated within this gated area was label as the percentage of ALDH+ cells.

Surface antigen analysis for Hsp90 was performed following standard antibody binding protocols. Briefly, cells were gently dissociated from the plates using Accutase and resuspended in PBS + 2% FBS at 5 × 10^5^ cells/mL. Cells were re-centrifuged in labeled microcentrifuge tubes and resuspended in 100 uL containing either 1:100 diluted Hsp90-PE antibody (LNCaP only) or 1:50 diluted Hsp90-PE antibody (ARCaPE, DU145, Myc-Cap). A corresponding tube with 1:50 unconjugated PE secondary antibody was used as a negative control. Cells were incubated in the dark on ice for 30 minutes before centrifugation and 1X wash with PBS + 2% FBS. 7-AAD was used a viability stain per manufacturer's recommendations (BD Biosciences). Cells were excited using the 488 nm laser and analyzed at 576 nm for the PE emission. Positively stained cells were gated out and sorted for downstream PCR analysis and the bottom 5% of non-stained cells were gated as a control population.

Analysis for GFP expression due to activation of the PSA promoter was performed in LNCaP and Myc-CaP cells using the vector provided by Dean Tang's lab [21, 98]. Vector transduction was confirmed in both the GFP positive and GFP negative populations via qPCR for GFP (Sense Primer: CTCGTGACCACCCTGACC, Antisense Primer: TAGTTGCCGTCGTCCTTGAA). Determination of surface bound Hsp90 was performed in transduced cells as described above. Gating strategy for analysis of surface-bound Hsp90 in transduced cells included auto-compensation to account for spectral overlap between GFP and PE emission spectra.

## SUPPLEMENTARY FIGURES


