# Supplementary material for: Secreted heat shock protein 90 promotes prostate cancer stem cell heterogeneity
Source: Oncotarget. 2016 Dec 27;8(12):19323–41. doi: 10.18632/oncotarget.14252 (PMC5386687; doi:10.18632/oncotarget.14252)
Supplement: Supplementary file 1 [file oncotarget-08-19323-s001.pdf]

Secreted heat shock protein 90 promotes prostate cancer stem cell heterogeneity

SUPPLEMENTARY FIGURES

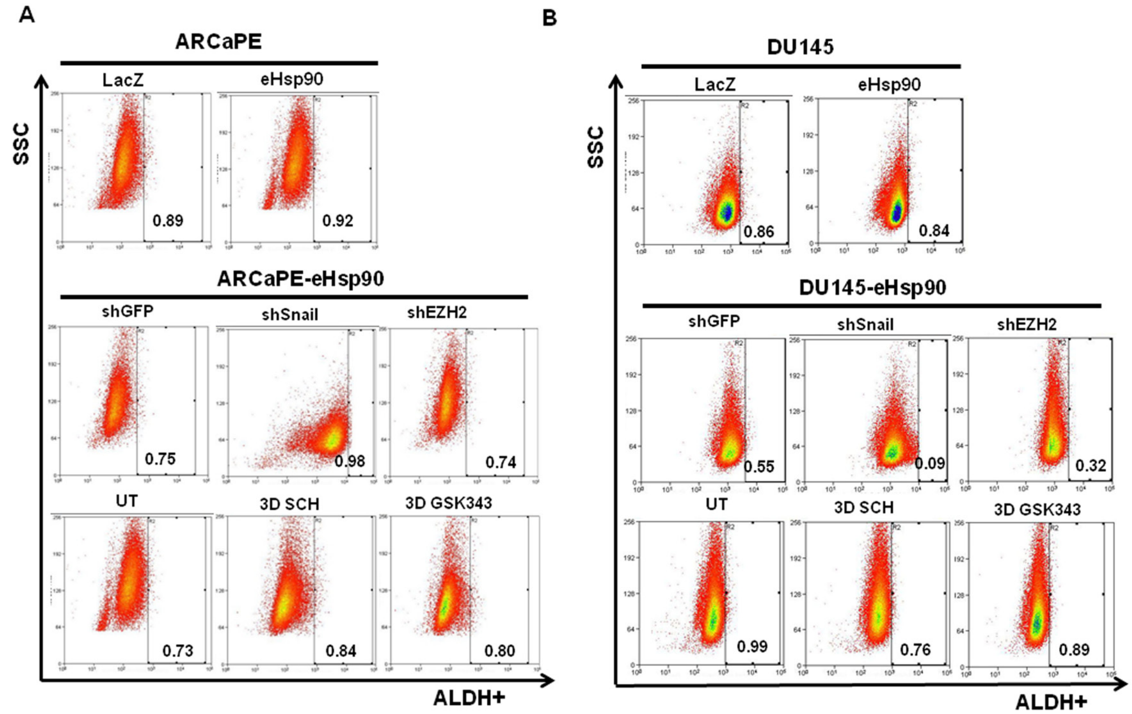

Supplementary Figure 1: DEAB Controls (for Figure 3). A, B. Representative flow cytometry scatter plots for the ALDEFLUOR assay generated for the ARCaPE-LacZ (A) or DU145 (B) models shown in Figure 3. Cells were treated with DEAB prior to and during sample incubation with ALDEFLUOR substrate. Numbers on the respective plots represent the percentage of ALDH+ events within the DEAB control samples matched to ALDEFLUOR samples depicted in Figure 3. Numerical values represent the percentage of ALDH+ events within the DEAB control samples matched to ALDEFLUOR samples.

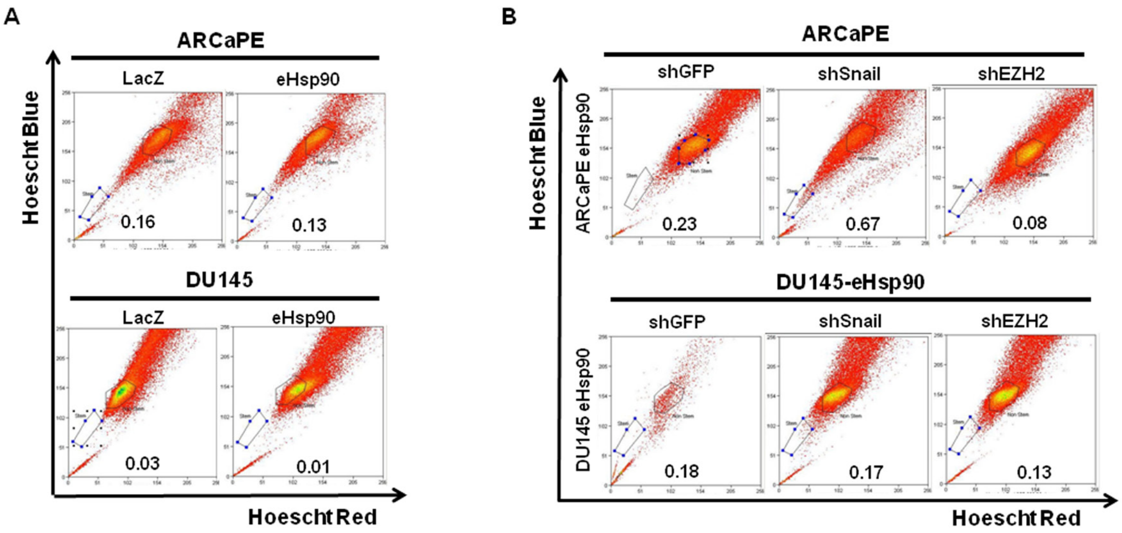

Supplementary Figure 2: Verapamil Controls (for Figure 4). A. Representative flow cytometry scatter plots for the side population for ARCaPE-LacZ relative to ARCaPE-eHsp90 and DU145-LacZ relative to DU145-eHsp90 upon treatement with 50 uM verapamil prior to and during sample incubation with Hoescht 33342 stain. Numerical values represent the percentage of side population positive events with the verapamil control samples matched to side population samples depicted in Figure 4A. Similar results were obtained for M12 (not shown) **B.** Representative flow cytometry scatter plots for the side population of the indicated ARCaPE-eHsp90 and DU145-eHsp90 derivative models, each treated with verapamil as in A.
